# Metabolic engineering of microbial cell factories for production of nutraceuticals

**DOI:** 10.1186/s12934-019-1096-y

**Published:** 2019-03-11

**Authors:** Shuo-Fu Yuan, Hal S. Alper

**Affiliations:** 1 Institute for Cellular and Molecular Biology, The University of Texas at Austin, Austin, TX USA; 2 McKetta Department of Chemical Engineering, The University of Texas at Austin, 200 E Dean Keeton St. Stop C0400, Austin, TX 78712 USA

**Keywords:** Metabolic engineering, Nutraceuticals, Value-added products, Co-culture system

## Abstract

Metabolic engineering allows for the rewiring of basic metabolism to overproduce both native and non-native metabolites. Among these biomolecules, nutraceuticals have received considerable interest due to their health-promoting or disease-preventing properties. Likewise, microbial engineering efforts to produce these value-added nutraceuticals overcome traditional limitations of low yield from extractions and complex chemical syntheses. This review covers current strategies of metabolic engineering employed for the production of a few key nutraceuticals with selecting polyunsaturated fatty acids, polyphenolic compounds, carotenoids and non-proteinogenic amino acids as exemplary molecules. We focus on the use of both mono-culture and co-culture strategies to produce these molecules of interest. In each of these cases, metabolic engineering efforts are enabling rapid production of these molecules.

## Background

Nutraceuticals are an important class of molecules that can exert long-term physiological benefits including preventing aging-associated diseases, depression, inflammation, arthritis, osteoporosis, gastrointestinal diseases, cardiovascular diseases, diabetes, and cancer. These molecules are traditionally isolated and sourced from plants (e.g. phytochemicals, carotenoids and vitamins), animals (e.g. polysaccharides), microorganisms (e.g. amino acids) and marine sources (e.g. glucosamine and very long-chain polyunsaturated fatty acids) [1–4] and have a global market of over $230 Billion in 2018 [5]. Growth in this area is certainly fueled by a renewed interest in the molecular underpinnings of more traditional medicine treatments [6]. However, long-term sustainability of these products, low overall abundances in plants [3], as well as a limited capacity to chemically modify these molecules for improved efficacy spur a new strategy for production—namely, a metabolic engineering approach [7, 8].

Metabolic engineering of nutraceutical products provides an attractive alternative to chemical synthesis and extraction that enables enantiomerically pure compounds to be produced at benign conditions without the requirement for high pressure and heat [3, 9]. In this regard, biosourced nutraceuticals can provide an environmentally-friendly process by using low-cost, non-food lignocellulosic feedstocks (such as agro-industrial and municipal wastes) to produce molecules of interest [8–10]. To accomplish these goals, a host of organisms have been explored including traditional microorganisms such as *Escherichia coli* and some GRAS (Generally Regarded as Safe) strains including *Saccharomyces cerevisiae* and *Corynebacterium glutamicum* [11–13]. These traditional hosts are typically selected on the basis of their well-described metabolism, safe use status for the pharmaceutical and food industries, and high genetic tractability [14]. Despite the prospects that metabolic engineering provides in this space, the usage of genetically modified organisms (GMOs) in commercial manufacturing processes requires regulation. In this regard, GMO regulation including approval processes, risk assessment, labeling, traceability, coexistence and membership in international agreements places additional demands on this field, which differ greatly across countries [15–17].

Beyond standard organisms, a variety of non-conventional GRAS microbes are being explored for nutraceutical production. As an example, the oleaginous organisms *Yarrowia lipolytica* [18–20] has been extensively studied for its innate ability to produce high quantities of lipid [21]. Using this capacity, DuPont has successfully rewired and commercialized *Y*. *lipolytica* for omega-3 polyunsaturated fatty acid production [22]. Finally, many nutraceuticals of interest are complex molecules (such as glycosides) that can be better produced via microbial co-cultures or synthetic consortia [23]. Thus, a co-culture strategy can diminish the metabolic burden on each microbial strain and thus enable a parallel construction of the optimized metabolic pathway in a modular fashion [24] while taking advantage of the favorable traits of each independent organism.

Through efforts in the engineering of each of these organisms (singly and in consortia), metabolic engineering efforts are enabling the high level production of many nutraceutical products. In this review, we highlight recent progress in the field. Specifically, we focus on recent efforts to increase production of polyunsaturated fatty acids, polyphenolic compounds, carotenoids and non-proteinogenic amino acids as exemplary nutraceutical molecules. While not comprehensive in describing every molecule with nutraceutical value, this review attempts to demonstrate the potential of using metabolic engineering strategies for sourcing this important class of molecules (Table 1).

**Table 1:** Production of nutraceuticals in engineered microorganisms from simple carbon sources

Product	Titer (mg/L)	Carbon source	Platform organism	Medium, fermentation type and parameters	References
Polyunsaturated fatty acids					
α-Linolenic acid	1400	Glucose	*Y. lipolytica* L36DGA1	YSC medium contained 80 g/L glucose; a pulse of 80 g glucose was added at 72 h/fed-batch 2 L bioreactor, 20 °C	[28]
EPA	56.6% in total lipids with 15% DCW^a^ 50% in total lipids with 25% DCW^a^	Glucose	*Y. lipolytica* ATCC 20362 *Y. lipolytica* Y4305	Nitrogen-rich medium contained 20 g/L glucose for the first stage fermentation, and nitrogen-limited medium contained 80 g/L glucose for the second stage fermentation/two-stage flask, 30 °C Two-stage 2 L bioreactor, 30 °C	[4] [22]
DHA	2.4 (5% of total fatty acids)	Complex media	*E*. *coli* DH5α	LB medium was supplemented with 1 mg/L cerulenin/flask, 15 °C after 1 mM IPTG induction	[32]
DHA	5.6% in total lipids	Glucose	*Y. lipolytica* Y4305	Cells were grown in MM medium containing 20 g/L glucose for 48 h, then transferred to HGM medium containing 80 g/L glucose for additional 72 h fermentation/two-stage fermentation, 30 °C	[31]
Polyphenols					
Naringenin	100.64	Glucose	*E. coli* BL21 (DE3)	MOPS medium contained 5 g/L glucose and 4 g/L NH_4_Cl/flask, 30 °C	[42]
Naringenin	54.4/112.9	Glucose	*S. cerevisiae* CEN.PK	Synthetic medium contained 20 g/L glucose and 10 g/L (NH_4_)_2_SO_4_/flask or batch 2 L bioreactor, 30 °C	[43]
Naringenin	21	Xylose	*E. coli* BW25113 and *S. cerevisiae* CEN.PK2-1C	Synthetic fermented medium contained 40 g/L xylose, 5 g/L yeast extract and inorganic salt/flask, 30 °C	[44]
Resveratrol	416 (glucose)/531 (ethanol)	Glucose or ethanol	*S. cerevisiae* CEN.PK102-5B	Medium contained 40 g/L glucose (for batch phase), trace metals and vitamin solutions; 16 g/L glucose or 17 g/L ethanol for feeding/fed-batch 1 L bioreactor, 30 °C	[47]
Resveratrol	812 (glucose)/755 (ethanol)	Glucose or ethanol	*S. cerevisiae* CEN.PK102-5B	Medium contained 40 g/L glucose (for batch phase), trace metals and vitamin solutions; 88 g/L glucose or 79 g/L ethanol for feeding/fed-batch 1 L bioreactor, 30 °C	[48]
Resveratrol	22.6	Glycerol	*E. coli* W3110 coculture	M9 medium contained 0.3 mM l-phenylalanine and 10 g/L glycerol/batch 1 L bioreactor, 30 °C	[49]
Kaempferol	27	Glucose	*S. cerevisiae* CEN.PK102-5B	Synthetic feed-in-time medium contained vitamins, dextrose polymer and enzyme minx/96-deep well plate, 30 °C	[54]
Quercetin	20				
Afzelechin	41	Glycerol	*E. coli* BL21star*™*(DE3)*ΔsucCΔfumC* (upstream strain) and BL21star™(DE3) (downstream strain)	Initial AMM medium contained 20 g/L glycerol; feed solution contained 2 × MOPS mix with 250 g/L glycerol/fed-batch bioreactor, 30 °C	[56]
Carotenoids					
Lycopene	1230	Glycerol	*E. coli* BL21(DE3)	M9 medium contained 40 g/L glycerol; engineered strain consumed around 130 g/L glycerol/fed-batch 150 L bioreactor, 30 °C	[71]
Lycopene	2370	Glucose or ethanol	*S. cerevisiae* CEN.PK2-1D	Feeding solution contained 500 g/L glucose and 15 g/L yeast extract for the first stage fermentation, and ethanol was used for the second stage fermentation/Two-stage fed-batch 7 L bioreactor, 30 °C	[64]
β-Carotene	3200	Glycerol	*E. coli* BL21(DE3)	Optimized medium contained 20 g/L glycerol; 400 g/L glycerol was fed at a rate of 3 g/L/h/fed-batch 5 L bioreactor, 34 °C after IPTG induction	[70]
β-Carotene	2100	Glycerol	*E. coli* ATCC 8739	Synthetic medium contained 10 g/L glycerol; 500 g/L glycerol was fed at a rate of 20 mL/h/fed-batch 7 L bioreactor, 37 °C	[72]
β-Carotene	4000	Glucose	*Y. lipolytica* MYA2613	Optimized medium with the C/N ratio at 3:1.5 for the first stage fermentation, and 600 g/L glucose was used for the second stage fermentation/Two-stage fed-batch 2 L bioreactor, 30 °C	[66]
β-Carotene	6500	Glucose	*Y. lipolytica* Po1d	YPD medium contained 20 g/L yeast extract, 40 g/L peptone and 5 g/L glucose; additional glucose was added after 6 h at a rate of 6 g/h/fed-batch 5 L bioreactor, 28 °C	[65]
Astaxanthin	432.8 (7.12 mg/g DCW)	Glycerol	*E. coli* W3110	Modified medium contained 30 g/L glucose and 5 g/L yeast extract (for batch phase); glycerol concentration was maintained at 0–2 g/L for feeding; 0.5 mM IPTG was added when OD_600_ reached 30–40/fed-batch 5 L bioreactor, 30 °C	[68]
Astaxanthin	217.9 (13.8 mg/g DCW)	Glucose	*S. cerevisiae* BY4742	YPD medium contained 20 g/L glucose; glucose feeding was controlled below 2 g/L and 30 g yeast extract was added every 12 h/fed-batch 5 L bioreactor, 30 °C	[67]
Astaxanthin	54.6 (3.5 mg/g DCW)	Glucose	*Y. lipolytica* GB20	YPD medium contained 80 g/L glucose/microtiter plate, 30 °C	[69]
Non-proteinogenic amino acid					
β-Alanine	32300	Glucose	*E. coli* W3110	Synthetic medium contained 20 g/L glucose and 9 g/L (NH_4_)_2_SO_4_; 240 g/L glucose was consumed/fed-batch 6.6 L bioreactor, 37 °C	[85]
GABA	4800	Glucose	*E. coli* BW25113	M9 medium contained 20 g/L glucose/flask, 37 °C	[87]
GABA	39000	Glucose	*C. glutamicum* ATCC 13032	GP1 medium contained 100 g/L glucose and 50 μg/L biotin; 498 g glucose was consumed/fed-batch 5 L bioreactor, 30 °C	[88]

^a^DCW represents EPA content at dry cell weight

## Polyunsaturated fatty acids

Polyunsaturated fatty acids (PUFAs) are essential fatty acids required for human development and health and are typically categorized into two major classes: omega-3 (n-3) and omega-6 (n-6) fatty acids with the ω-3 fatty acids being the major focus of most industrial microbial engineering and hence the focus in this review. These molecules play an important role on health including in the areas of development of the nervous system, cardioprotective functions, reducing the risk of neurodegenerative and inflammatory diseases, promoting the reduction of triglyceride contents in the serum as well as preventing cancer [1, 25, 26]. Commercially important ω-3 fatty acids include α-linolenic acid (ALA; C18:3n − 3), eicosapentaenoic acid (EPA; C20:5n − 3) and docosahexaenoic acid (DHA; C22:6n − 3). Given the increased recognition of health benefits from these molecules, demand for ω-3 PUFAs is growing and expected to reach a global demand of 241 thousand metric tons with a value of $4.96 billion by the year 2020 [27]. However, traditional sourcing of these molecules has been restricted to low productivity and unsustainable processes including extraction of ALA from plant seeds and EPA as well as DHA from fish oils [4, 28]. As a result, the use of rewired microbes to produce these PUFAs could provide an alternative approach that is both economically viable and sustainable.

### EPA and DHA

EPA and DHA biosynthesis is typically pursued through the aerobic desaturase/elongase pathway although production is feasible through an anaerobic polyketide synthase (PKS) pathway [4]. DuPont researchers used this aerobic pathway in *Y. lipolytica* to generate a strain capable of producing EPA at 56.6% of the total fatty acids and about 15% of the dry cell weight, a value that is the highest percentage among known EPA sources [4]. This same group later developed a new commercial strain (*Y. lipolytica* Z5567) that optimized carbon flux toward EPA biosynthesis pathway, eliminated β-oxidation and fine-tune regulated EPA transportation [22, 29]. When cultivated using a two-stage fed-batch fermentation process (using nitrogen-rich medium for growth phase and nitrogen-limiting conditions for oil production), this strain was capable of producing an oil comprising EPA at 50% and 25% dry cell weight [22, 29].

Using this PUFA-production technology, two commercial products, New Harvest™ EPA oil and Verlasso^®^ sustainably farmed salmon, have been developed [29]. Beyond this example, several alternative hosts and technologies exist for the production of DHA including numerous marine microalga strains due to their innate high content of DHA (30–40% of total fatty acids) [30]. While this is the case, more traditional hosts, such as bacteria and yeast are often more limited with respect to DHA production (less than 6% of total fatty acids) [31, 32], thus we will not discuss production in these hosts here.

### ALA

**Fig. 1:**
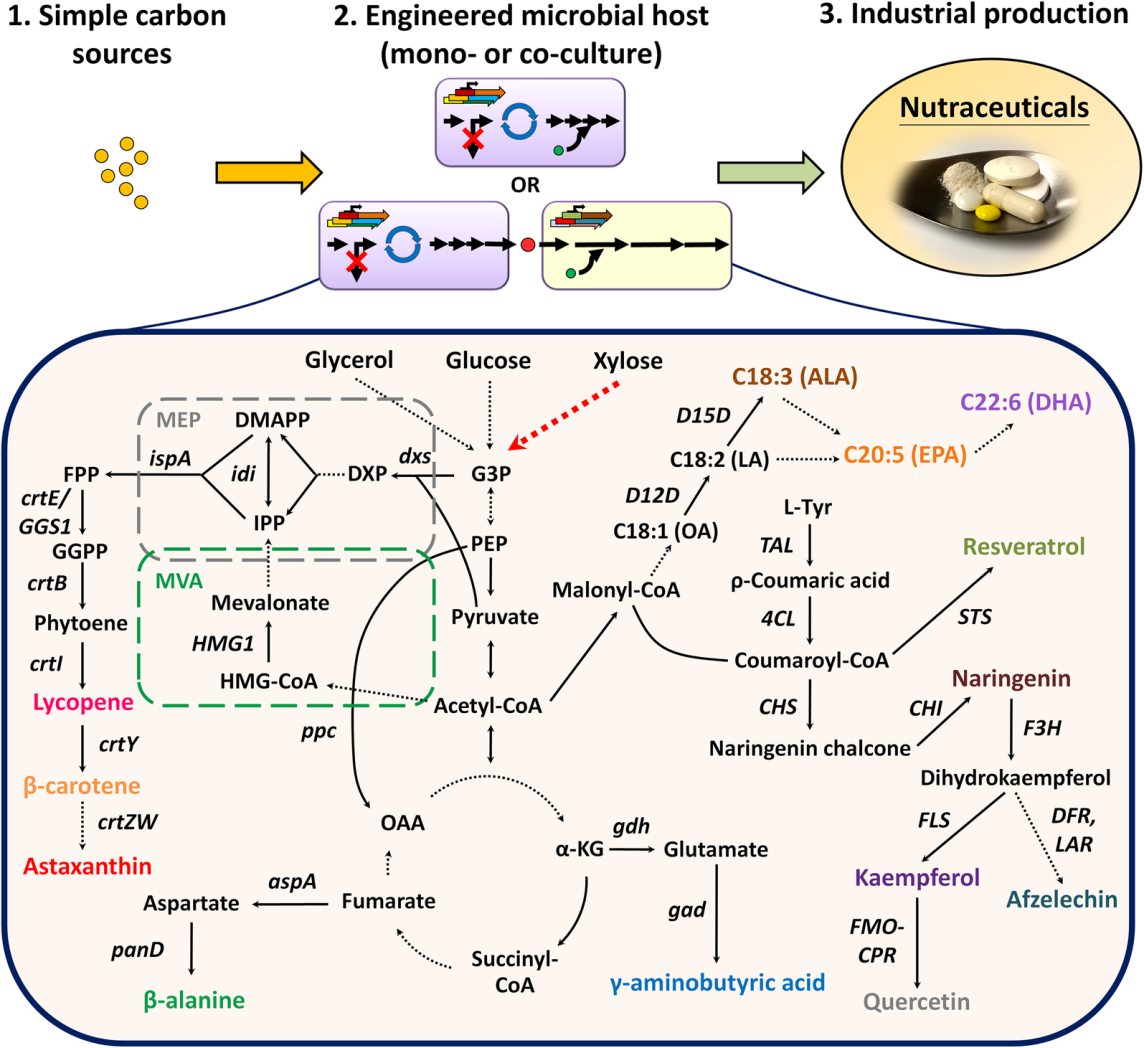
Microbial-based production of nutraceuticals from simple carbon sources. A solid line is enzymatic reaction through an indicated enzyme and a dashed line represents reaction involving multiple enzymes. Xylose utilization pathway absent in *S. cerevisiae* or existing in *Y. lipolytica* but poorly expressed is labeled with red dashed arrow. Enzymes encoded by the genes shown are dxs, DXP synthase; idi, IPP isomerase; ispA, FPP synthase; HMG1, 3-hydroxy-3-methyl-glutaryl-coenzyme A reductase; crtE or GGS1, geranylgeranyl pyrophosphate synthase; crtB, phytoene synthase, crtI phytoene desaturase; crtY, lycopene cyclase; crtW, β-carotene ketolase; crtZ, β-carotene hydroxylase; ppc, phosphoenolpyruvate carboxylase; aspA, aspartase; panD, l-aspartate-α-decarboxylase; gdh, glutamate dehydrogenase; gad, glutamate decarboxylase; D12D, Δ12-desaturase; D15D, Δ15-desaturase; TAL, tyrosine ammonia lyase; 4CL, 4-coumarate:CoA ligase; CHS, chalcone synthase; CHI, chalcone isomerase; STS, stilbene synthase; F3H, flavanone 3-hydroxylase; FLS, flavonol synthase; FMO, flavonoid 30-monooxygenase; CPR, cytochrome P450 reductase; DFR, dihydroflavonal 4-reductase; LAR, leucoanthocyanidin reductase. Abbreviations of chemicals are G3P, glyceraldehyde 3-phosphate; DXP, 1-deoxy-D-xylulose-5-phosphate; IPP, isopentenyl pyrophosphate; DMAPP, dimethylallyl pyrophosphate, FPP, farnesyl pyrophosphate; L-Tyr, l-tyrosine; PEP, phosphoenolpyruvate; α-KG, 2-oxoglutarate; OAA, oxaloacetate; OA, oleic acid; LA, linoleic acid; ALA, -linolenic acid; EPA, eicosapentaenoic acid

An additional ω-3 fatty acid, α-linolenic acid (ALA), has been explored also in the oleaginous yeast *Y. lipolytica*. Biosynthesis of ALA requires a Δ15-desaturase to convert native unsaturated fatty acids of oleic acid (C18:1n − 9) and linoleic acid (C18:2n − 6) into the ALA [33] (Fig. 1). Using a previously engineered strain of *Y. lipolytica* that can produce nearly 80% of lipids as an unsaturated C18 s [34], it was possible to create a platform for ALA biosynthesis [28]. Specifically, heterologous expression of a codon-optimized, bifunctional Δ12/Δ15-desaturase from *R. kratochvilovae* coupled with a low-temperature fermentation (20 °C) produced significantly increased ALA content. The resulting strain was capable of producing ALA to upwards of 30% of total fatty and achieving titers of 1.4 g/L ALA in fed-batch fermentation, the highest reported titer in a yeast host [28]. Collectively, these results highlight the use of microorganisms (especially oleaginous yeasts) for the production of nutritional fatty acids.

## Polyphenols

Polyphenolic compounds (including flavonoids, isoflavonoids and stilbenoids) are secondary metabolites (typically in plants) that protect against stress conditions such as ultraviolet radiation, pathogenic infection and physical damage. Likewise, these polyphenol molecules can have similar health-promoting benefits in humans including providing strong antioxidant and anti-inflammatory activities as well as help in the prevention of obesity, diabetes, hypertension, neurodegenerative diseases, cancer and metabolic syndromes [1, 2, 35, 36]. Given these varied and valuable uses, the global market is expected to reach $1121 million by 2022 [37]. Biochemically, the diverse structure of plant-derived polyphenols originate from the aromatic amino acids phenylalanine (Phe) or tyrosine (Tyr) (Fig. 1). Thus, strain engineering efforts as describe below are focused both on increasing this flux and complementing essential plant-based enzymes. This review focuses mostly on progress toward producing the molecules naringenin, resveratrol, and other similar flavonoids.

## Naringenin

Naringenin is the central precursor of most flavonoids, yet still has its own bioactivity with respect to anti-diabetic, antioxidant, antiapoptotic and neuro-protective properties [36, 38]. Biosynthesis of this molecule requires two components: formation of the starter element of *p*-coumaric acid followed by sequential condensations with malonyl-CoA. Thus, strain engineering efforts have revolved around both of these steps.

First, the supply of *p*-coumaric acid in the cell can be achieved via two separate biosynthetic routes. In one pathway, the activity of phenylalanine ammonia lyase (PAL) can convert pheylanlanine into cinnamic acid, which can be further hydroxylated by a cytochrome P-450-dependent cinnamate-4-hydroxylase (C4H) to yield *p*-coumaric acid [36]. This route is particularly challenging for bacterial cells where heterologous P450 expression typically suffers (and where reductase partners are missing) [39]. The alternative pathway relies upon direct conversion of tyrosine into *p*-coumaric acid via a tyrosine ammonia lyase (TAL) [40]. After either approach, the resulting *p*-coumaric acid is then converted into its corresponding coenzyme A ester, coumaroyl-CoA, through the activity of a 4-coumarate:CoA ligase (4CL) [41].

Second, three molecules of malonyl-CoA are subsequently condensed with the molecule of coumaroyl-CoA by a chalcone synthase enzyme (CHS, a type III polyketide synthase) to form the molecule naringenin chalcone. Finally, this compound is converted into naringenin through either the action of a chalcone isomerase (CHI) or via a non-enzymatically catalyzed reaction [36, 39] (Fig. 1).

For the case of all common host microorganisms described above, these two pathways are established via heterologous expression. Likewise, the supplementation of precursor metabolites including the relatively expensive and low-water-solubility *p*-coumaric acid or tyrosine is not feasible for industrial production. As a result, strain engineering has been used to create de novo production platforms for molecules like naringenin. To this end, modular pathway optimization, combinatorial tuning of TAL, 4CL, CHS, CHI enzymes using modified plasmid gene copy numbers and inducible promoter strengths, and enhancement of the supply of intracellular tyrosine via a feedback resistant tyrA^fbr^-aroG^fbr^ cassette enabled *E. coli* to produce around 100 mg/L of naringenin directly from glucose [42]. In a similar fashion, co-expression of the naringenin biosynthesis genes (*PAL1*, *C4H*, *CPR1*, *4CL3*, *CHS3* and *CHI1* from *A. thaliana* as well as *CHS3* and *TAL1* from *R. capsulatus*) and alleviation of competing pathway (elimination of phenylpyruvate decarboxylase activity and tyrosine feedback inhibition) resulted in de novo production of naringenin in *S. cerevisiae* at titers of around 54 mg/L and 113 mg/L from glucose in flask and a controlled 2-L fermenter, respectively [43].

As an alternative approach, a synergistic co-culture system was recently developed for the production of naringenin from xylose [44]. In this scheme, the biosynthetic pathway for naringenin was split such that tyrosine-producing *E. coli* and naringenin-producing *S. cerevisiae* were made to be a synergistic community. To establish a stable community, *E. coli* utilized the xylose and excreted a growth inhibiting acetate, while *S. cerevisiae* utilized acetate as the carbon source without producing ethanol. Through optimizing the ratio of inoculum size and cell ratios, a titer of 21 mg/L naringenin was obtained in co-culture, representing a nearly eightfold increase over that of the mono-culture of yeast [44].

### Resveratrol

Related to naringenin, the stilbene resveratrol (commonly found in red wine) has been of increasing interest as an antioxidant and anti-inflammatory agent as well as putative associations with longevity. Functionally, resveratrol is a phytoestrogen receptor agonist that can suppress expression of cyclooxygenase-2 (COX-2), an enzyme promotes tumor growth, and has been seen to have a role in preventing cardiovascular and neurodegenerative disease [45, 46]. Biochemically, production of this stilbene derives from the same *p*-coumaric acid precursor described above for naringenin. Subsequently, a stilbene synthase (STS, a type III polyketide synthase) converts the coumaroyl-CoA into a stilbene via three molecules of malonyl-CoA (Fig. 1).

Similar to naringenin, a variety of approaches have been explored for the microbial production of resveratrol. Resveratrol can be directly biosynthesized from cheap carbon sources via the tyrosine pathway for *p*-coumaric acid-production in *S. cerevisiae* leading to up to 531 mg/L of resveratrol in fed-batch fermentation [47]. This strain was achieved through integration of the resveratrol biosynthetic genes (*TAL* from *H. aurantiacus*, *4CL1* from *A. thaliana* and *VST1* from *V. vinifera*) as well as introduction of the *ARO4*^*K229L*^ (feedback-inhibition resistant DAHP synthase), *ARO7*^*G141S*^ (feedback-inhibition resistant chorismate mutase) and *ACC1*^*S659A, S1157A*^ (inactivation-resistant acetyl-CoA carboxylase) mutants with multiple chromosomal copies [47]. The same research group also demonstrated de novo resveratrol synthesis from glucose or ethanol via the phenylalanine pathway in *S. cerevisiae* [48]. This later feat was accomplished via increases in phenylalanine and malonyl-CoA supply, copy number enhancement of the resveratrol pathway genes and P450 activity (overexpression of cytochrome P450 reductase (ATR2) from *A. thaliana* and cytochrome B5 (CYB5) from *S. cerevisiae*). The resulting strains of yeast were able to produce 812 and 755 mg/L resveratrol from glucose and ethanol feed, respectively, in fed-batch fermentation. Finally, an *E. coli*-*E. coli* co-culture approach has been demonstrated employing two engineered *E. coli* to produce resveratrol from glycerol [49]. In this scheme, the first strain utilizes glycerol as a carbon source to synthesize *p*-coumaric acid and excrete it to the medium to be transported into the second engineered *E. coli* strain rewired to efficiently produce malony-CoA and the STS enzyme to produce titers of 22.6 mg/L of resveratrol [49].

### Other flavonoids

Several additional flavonoids of nutraceutical interest have been explored with microbial production. For example, kaempferol and quercetin are commonly explored based on exhibited anti-cancer, cardio-protective and anti-inflammatory effects [50–53]. Likewise, kaempferol is exhibited to inhibit cancer cell growth and lead to cancer cell apoptosis [51]. In this regard, animal studies have demonstrated its protective role against doxorubicin (DOX)-induced cardiotoxicity [50]. Quercetin is also seen as a potent anti-cancer molecule able to suppresses the growth and invasive/metastatic potential of B16-BL6 melanoma cells in mice [53] primarily by a reduction in reactive oxygen species (ROS) levels [52]. Biochemically, kaempferol is derived from the precursor naringenin via the activity of a flavanone 3-hydroxylase (*F3H*) and flavonol synthase (*FLS*) [36] (Fig. 1). Complete, de novo production of kaempferol from glucose was recently reported by overexpression of F3H from *A. mongholicus* and FLS from *A. thaliana* in a naringenin-producing *S. cerevisiae* [54]. The resulting titer of 27 mg/L exceeded previous reported titers, but also demonstrated a limited enzymatic capacity as 11 mg/L of *p*-coumaric acid accumulated. The same research group also introduced a cytochrome P450 flavonoid monooxygenase (*FMO*), which was fused in-frame to the cytochrome P450 reductase (*CPR*) from *C. roseus*, into this kaempferol-producing strain to produce quercetin [54]. The resulting strain produced around 20 mg/L of quercetin from glucose, the highest extracellular concentration reported to date.

A final class of flavonoids, the flavan-3-ols, are sought after for their ability to reduce the risk of cardiometabolic disorders [55]. Flavan-3-ols can be biosynthesized from naringenin via the actions of flavanone 3-hydroxylase (*F3H*), dihydroflavonal 4-reductase (*DFR*) and leucoanthocyanidin reductase (*LAR*) (Fig. 1). An optimized *E. coli* co-culture system was recently deployed to achieve high titers of afzelechin, a flavan-3-ol monomer, from glycerol [56]. To accomplish this, the complete afzelechin pathway was partitioned into the malonyl-CoA requiring upstream module (coumaric acid to naringenin) and the NADPH requiring downstream module (naringenin to afzelechin). This co-culture system had a 970-fold improvement in afzelechin titer over previously reported mono-culture production schemes. Following optimization of fermentation parameters such as strain compatibility, carbon source, temperature, induction time point and inoculation ratio, the finalized co-culture system produced around 41 mg/L of afzelechin [56]. These results show the promise of co-culture systems, especially for the production of more complex natural products [24].

## Carotenoids

Carotenoids are naturally occurring, lipid-soluble pigments that are well sought after in the field both for their capacity as natural colorants and for their antioxidant properties [57]. As antioxidants, these molecules have nutraceutical benefits and preventive effects against oxidative damage- and inflammation-related diseases such as cancer, cardiovascular diseases, atherosclerosis, neurodegenerative disorders, and diabetes [58–60]. As such, the global market for carotenoids is expected to reach $2.0 billion by 2022 [61].

Heterologous production of value-added carotenoids, including lycopene, β-carotene, and astaxanthin, in non-carotenogenic microorganisms has been extensively studied [62–69]. Biochemically, carotenoids are derived from two building blocks, isopentenyl pyrophosphate (IPP) and dimethylallyl pyrophosphate (DMAPP), that can be synthesized by either the mevalonic acid (MVA) pathway or methylerythritol 4-phosphate (MEP) pathway [70]. In *E. coli*, overexpression of two major rate-limiting native enzymes DXP synthase (Dxs) and IPP isomerase (Idi) increases the supply of IPP and DMAPP leading to an increase in farnesyl diphosphate (FPP) by GPP/FPP synthase (IspA) [62]. In contrast, FPP is synthesized via native MVA pathway in eukaryote system [57]. The FPP is then converted into lycopene via the introduction of a heterologous pathway containing geranylgeranyl pyrophosphate synthase (CrtE), phytoene synthase (CrtB) and phytoene desaturase (CrtI). In a similar fashion, β-carotene can be produced from lycopene through the overexpression of a lycopene cyclase (CrtY), and astaxanthin can be biosynthesized from β-carotene via overexpression of β-carotene ketolase (CrtW or BKT) as well as β-carotene hydroxylase (CrtZ) (Fig. 1).

An engineered *S. cerevisiae* has been recently developed to improve capacity for lycopene production [64]. This rewiring was achieved through systematic metabolic engineering including overexpression of enzymes for redirecting carbon flux from ethanol to acetyl-CoA and mevalonate precursors, increasing cofactor NADPH generation as well as integration of lycopene biosynthetic pathway (*crtE* from *T. x media*, *crtB* from *P. agglomerans* and *crtI* from *B. trispora*). Coupling with optimized triacylglycerol (TAG) metabolism by overexpressing a fatty acid desaturase *OLE1* (increasing unsaturated fatty acid supply) and deletion of Seipin complex gene FLD1 (regulating lipid-droplet size), the optimal strain produced 2.37 g/L lycopene in a two-stage fed-batch fermentation (with the first stage used for biomass accumulation and the second stage for producing lycopene from fed ethanol). The researchers achieved the highest content (73.3 mg/g dry cell weight) reported to date in oleaginous microbes or in *S. cerevisiae*, which could potentially replace the natural producer *B. trispora* in industrial production. Despite the FLD1-knockout phenotype showing a decrease in the cell mass, this study demonstrated modulating the lipid-droplet size and composition was efficient in promoting lycopene accumulation [64].

In a separate study, the introduction of a heterogenous MVA pathway has been pursued as an alternative strategy for carotenoids overproduction in *E. coli*. In this regard, a highly efficient lycopene-producing *E. coli* was constructed through targeted engineering strategy to leverage the MVA pathway and introduction of lycopene pathway with extra copies of the *idi* gene, leading to a titer of 1.44 g/L lycopene from glycerol in fed-batch fermentation. This strain was also successfully scaled to the 100 L fed-batch fermentation level achieving a titer of 1.23 g/L [71].

An additional carotenoid of strong interest is β-carotene. Engineering in *E. coli* led to 3.2 g/L in a glycerol fed-batch experiment with a strain with improved MEP pathway containing isopentenyl pyrophosphate isomerase (FNI) from *B. subtilis* and geranyl diphosphate synthase (GPPS2) from *A. grandis* [70]. To do so, this study used exogenous expression of *A. grandis* GPPS2 as well as glycerol as a carbon source to reduce acetic acid accumulation [70]. A complementary approach also in *E. coli* optimized metabolic modules of β-carotene synthesis (CrtEXYIB from *P. agglomerans*), MEP (overexpression of Dxs and Idi) and two central metabolic modules (TCA cycle and pentose phosphate (PPP) pathway) to enable 2.1 g/L β-carotene in fed-batch fermentation [72]. Moreover, this study suggested that increasing NADPH supply was more important than ATP for improving carotenoid production [72].

While carotenoid production in bacteria is successful, prior results demonstrated a nexus between yeast and their capacity to store molecules like carotenoids in lipid droplets [64–66]. To this end, the fine-tune expression of enzymes (native enzymes for increasing supply of geranylgeranyl diphosphate (GGPP) precursor as well as exogenous enzymes carRP and carB from *Mucor circinelloides* for conversion of GGPP into β-carotene) using strong promoters and sequential multiple-copy integration led to a strain of *Y. lipolytica* capable of producing 4 g/L β-carotene in a fed-batch fermentation process with a nitrogen-limited medium [66]. Additionally, an engineered *Y. lipolytica* β-carotene overproducer was recently developed via direct rewiring of flux toward acetyl-CoA along with overexpression of downstream geranylgeranyl diphosphate synthase (GGS1), *M*. *circinelloides* carRP and carB under the control of strong TEF promoter. This strain was able to produce 6.5 g/L of β-carotene with a content of 89.6 mg/g dry cell weight in a fed-batch fermentation [65]. Collectively, these results highlight the ability to achieve g/L titers of bioactive carotenoids.

In recent years, high astaxanthin production in *E. coli* and yeasts have been achieved. Metabolic engineering of *E. coli* through overexpression of heterologous *crt* genes (crtEYIBZ) from *P. ananatis* and *ispDF* in DXP pathway resulted in 433 mg/L of astaxanthin (equivalent to 7.12 mg/g DCW) with a high productivity (9.62 mg/L/h) in glycerol fed-batch fermentation [68]. Despite the use of an industrially-limiting inducer, IPTG, employed in this study, the astaxanthin titer and productivity achieved with the engineered *E. coli* is the highest reported to date. One limitation in astaxanthin production is the general promiscuity of bacterial CrtWs and CrtZs enzymes that leads to diverse carotenoid intermediate profiles. To address this limitation, a recent study combined metabolic engineering and directed evolution to enhance astaxanthin production and ratio in *S. cerevisiae* [67]. To accomplish this, the CrtZ gene from *A. aurantiacum* and CrtW gene from *B. vesicularis* was introduced into a high β-carotene producer, SyBE_Sc118030, and subjected to atmospheric and room temperature plasma (ARTP) mutagenesis to ultimately obtain a strain capable of producing astaxanthin at a titer of 217.9 mg/L with the highest reported yield (13.8 mg/g DCW) in a fed-batch fermentation. Moreover, this study led to the discovery of three additional gene targets critical for product formation and regulation (*CSS1*, *YBR012* *W*-*B* and *DAN4*).

Another example of using a GRAS yeast to produce value-added astaxanthin was recently demonstrated in the oleaginous yeast *Y. lipolytica* [69]. To do so, introduction of a β-carotene biosynthesis pathway along with optimization of upstream MVA pathway (HMG1 and GGS1), downregulation of the competing squalene synthase *SQS1*, and overproduction of bacterial enzymes involved in astaxanthin synthesis (β-carotene ketolase crtW from *Paracoccus* sp. N81106 and β-carotene hydroxylase crtZ from *P. ananatis*) led to 10.4 mg/L astaxanthin. To redirect metabolic flux towards astaxanthin production, an additional effort in optimizing the copy numbers of *crtZ* and *crtW* resulted in boosting astaxanthin titer to 54.6 mg/L (3.5 mg/g DCW) in a microtiter plate cultivation. Notably, this study reported the first engineering of *Y. lipolytica* for this product and identified the role of β-carotene hydroxylase (*crtZ*) as a critical step in conversion of β-carotene into astaxanthin.

## Non-proteinogenic amino acid

Non-proteinogenic amino acids are a class of amino acids that are widely found in nature and are not incorporated into natural proteins. They are generally utilized as intermediates in primary metabolic pathways or building blocks for small bioactive peptide scaffolds [73]. Despite not being used in the biosynthesis of any protein, some small molecules such as β-alanine or γ-aminobutyrate (GABA) exhibit physiological benefits in humans [74, 75]. β-alanine (or 3-Aminopropionic acid), the simplest β-amino acid, is a structural intermediate between neurotransmitters glycine (α-amino acid) and GABA (γ-amino acid). Additionally, this molecule servers as a precursor for the biosynthesis of pantothenic acid (vitamin B_5_), an essential a component of coenzyme A [74]. Besides, this molecule has grown in interest as a sport supplement ingredient as it serves as the rate-limiting precursor of carnosine (β-alanyl-l-histidine), a dipeptide that buffers exercise-induced metabolic acidosis [74, 76, 77]. As a result, the global β-alanine market was valued at $64 million in 2017 and projected to reach $91 million by 2025 [78]. GABA is the main inhibitory neurotransmitter in the human central nervous system and studies have demonstrated its potential as an anti-diabetic, anti-hypertensive, relaxation and immunity enhancing molecule [75, 79–81]. As a result, this molecule has recently become a widely available food supplement with an anticipated global market of $64 million by 2025 [82].

### β-Alanine

Biochemically, β-alanine is formed through the action of l-aspartate-α-decarboxylase (PanD or ADC) that catalyzes the decarboxylation of l-aspartate (Fig. 1). Although a recent study reported a successful enzymatic conversion via ADC with a high efficiency (97.2%), the method was expensive as it required both the precursor l-aspartate and large quantities of enzyme [83]. Hence, de novo microbial production of β-alanine from cheap carbon sources offers a great potential for the industry.

Current efforts for increasing β-alanine production still focus on bacterial hosts as fungal organism (such as *S. cerevisiae*) do not have orthologs of *panD* or innately high fluxes toward precursors [84]. A novel metabolic pathway has been recently designed for the production of β-alanine from glucose in *E. coli* to produce 32.3 g/L, the highest reported titer to date [85]. To do so, this strain utilized an overexpression of *C. glutamicum* PanD (an enzyme possessing much higher *specific* activity than the *E. coli* counterpart) and aspartase (AspA) as well as phosphoenolpyruvate carboxylase (Ppc) in a highly fumaric acid-producing *E. coli*.

### GABA

Biochemically, GABA can be synthesized by decarboxylation of L-glutamate via glutamate decarboxylase (GAD) (Fig. 1) and its overproduction in microbial hosts have been explored. One particular challenge for the production of GABA was the acidic conditions required for activity of the *E. coli* GAD enzyme. However, recent advances obtained an *E. coli* GadB mutant (Glu89Gln/Δ452–466) through rational mutagenesis that had a broadened pH range up to 7 [86]. The incorporation of this GadB mutant into a dynamically controlled cell that contains a GABA production unit (bypass for precursor metabolite supply and upregulation of GABA transporter) along with a cell growth control unit (interruption of the TCA and glyoxylate cycles), it was possible to produce 4.8 g/L of GABA from glucose [87]. An alternative strategy for production utilized *C. glutamicum* as a platform host owing to its high production of L-glutamate, the direct precursor of GABA. A recombinant *C. glutamicum* strain was created expressing this same *E. coli* GadB mutant (Glu89Gln/Δ452–466) and led to a titer of 39 g/L with a productivity of 0.536 g/L/h in fed-batch fermentation [88]. This fermentative process (72 h cultivation) greatly shortens GABA fermentation time compared with previous reports (96–168 h) and demonstrates the potential to make this molecule at high titers and rates.

## Conclusions

A large array of nutraceutical products are being explored through the use of metabolic engineering. This approach bypasses the traditional challenges of direct extraction from animals and plants and provides an environmentally-friendly and sustainable platform for industrial production. As demand for these products continuously increase along with population growth, the use of metabolic engineering becomes more important. Likewise, the use of modular co-culture engineering is also an emerging approach with significant advantages for the production of nutraceuticals, especially those that build from more complex precursors. Continued advances in both synthetic biology and basic genetic engineering are increasing the type and number of available host organisms to meet demands for current and future nutraceutical products.
